# Nutritional status of pre-school children from low income families

**DOI:** 10.1186/1475-2891-10-43

**Published:** 2011-05-08

**Authors:** Denise O Shoeps, Luiz Carlos de Abreu, Vitor E Valenti, Viviane G Nascimento, Adriana G de Oliveira, Paulo R Gallo, Rubens Wajnsztejn, Claudio Leone

**Affiliations:** 1 Departamento de Saúde Materno-infantil, Faculdade de Saúde Pública, Universidade de São Paulo (USP), São Paulo, SP, Brasil; 2 Departamento de Morfologia e Fisiologia, Faculdade de Medicina do ABC, Santo André, SP, Brasil; 3 Departamento de Medicina, Disciplina de Cardiologia, Universidade Federal de São Paulo (UNIFESP), São Paulo, SP, Brasil; 4 Departamento de Patologia, Faculdade de Medicina, Universidade de São Paulo, São Paulo, SP, Brasil

## Abstract

**Background:**

We evaluated growth and nutritional status of preschool children between 2 and 6 years old from low income families from 14 daycare centers.

**Methods:**

Cross-sectional study with 1544 children from daycare centers of Santo Andre, Brazil. Body weight (W), height (H) and body mass index (BMI) were classified according to the 2000 National Center for Health Statistics (CDC/NCHS). Cutoff points for nutritional disorders: -2 z scores and 2.5 and 10 percentiles for malnutrition risk, 85 to 95 percentile for overweight and above BMI 95 percentile for obesity. Stepwise Forward Regression method was used including age, gender, birth weight, breastfeeding duration, age of mother at birth and period of time they attended the daycare center.

**Results:**

Children presented mean z scores of H, W and BMI above the median of the CDC/NCHS reference. Girls were taller and heavier than boys, while we observed similar BMI between both genders. The z scores tended to rise with age. A Pearson Coefficient of Correlation of 0.89 for W, 0.93 for H and 0.95 for BMI was documented indicating positive association of age with weight, height and BMI. The frequency of children below -2 z scores was lower than expected: 1.5% for W, 1.75% for H and 0% for BMI, which suggests that there were no malnourished children. The other extremity of the distribution evidenced prevalence of overweight and obesity of 16.8% and 10.8%, respectively.

**Conclusion:**

Low income preschool children are in an advanced stage of nutritional transition with a high prevalence of overweight.

## Background

Several countries are going through a period of epidemiological transition with reduced incidence of infectious diseases, child mortality and birth rates, associated with high prevalence of chronic diseases and increase in life expectancy at birth [1]. With respect to nutritional transition this situation also results in decreased prevalence of malnutrition in childhood and improved growth pattern of children, a fact that was also observed recently in Brazil [2-4]. In the last two decades in industrialized countries this trend has continued with a higher prevalence of obesity and its consequences, presenting in adults from higher socio-economic classes [5].

Since the early 90's several authors have noted the obesity epidemic affecting developing countries as this change in nutritional status is occurring much faster than in developed countries [3,4,6,7]. The obesity epidemic quickly spread to teenagers, school age children and recently to preschool children, mainly three years old [7]. As a result of early obesity onset several other health problems may arise in childhood, including hypertension, dyslipidemia, type 2 diabetes and cardiovascular problems, which may impair quality of life and decrease life expectancy [8,9].

The literature has already demonstrated the relationship between childhood poverty and abdominal obesity in adulthood [10]. A rise in inactivity is also a risk factor for obesity [11,12]. As mentioned above, obesity is a risk factor for many diseases such as certain cancers, hypertension, type II diabetes mellitus, dyslipidemia, metabolic syndrome and coronary heart disease [13,14]. The primary cause of malnutrition in developed countries is disease. Disease related malnutrition is associated with adverse effects on clinical outcome. These adverse effects vary from impaired wound healing and postoperative complications to mortality. Poor nutritional status has not only been associated with in-hospital adverse effects, but also with adverse effects at both pre-admission and post-discharge [15,16].

According to the IDB (Indicadores de Dados Básicos - Indicators of Basic Data), in 2008 the percentage of poor people in the State of São Paulo was 14.9% [17]. Data of overweight and obesity rates in Brazilian school-age children between 2 and 6 years old from low income families are limited. Because country-specific studies do not provide generalizable information, the World Health Organization (WHO) has highlighted the need to study child obesity around the world [18]. In addition, new evidences regarding overweight prevalence in children from low income families are important to direct and implement public politics and improve public health system. Thus, given the relevance of obesity in infancy and the lack of studies which investigate its association with socioeconomic status, we endeavored to evaluate growth and nutritional status of children from low income families. We also evaluated the relationship between obesity/overweight and child age, gender, birth weight and duration of breast feeding.

## Methods

This is a cross-sectional study of anthropometric data in preschool children from 14 daycare centers linked to the Department of Health and the Federation of charities (FEAS) in Santo Andre, SP, Brazil. The data collection was performed between 2001 and 2002. Santo Andre is a city with approximately 600,000 inhabitants and a total of 43 daycare centers. The Department of Health and the FEAS develop systematic surveillance activities to children's health enrolled in day care centers. The project was undertaken in partnership between the day care centers and the public system. This study was explained to the parents or guardians responsible for the child and it was begun only after his or her consent according to the standards of the committees of research and ethics. All experimental procedures were in compliance with the Helsinki Declaration. The study was approved by the Ethics Committee in Research of the Faculdade de Medicina da Universidade de São Paulo (FMUSP, number 923/00).

The study population included 1639 children aged between 2 and 6 years old, all from low income families. We excluded 95 (5.8%) because they were not of preschool age, hence, it remained 840 (54.4%) boys and 704 (45.6%) girls from a total of 1544 children (Table 1). We also excluded children who presented congenital diseases, history of nutritional and metabolic diseases, chronic diseases that could influence their growth and those who did not agree to collect data. This information was collected during the interview. According to the government (http://www2.santoandre.sp.gov.br/), this population represents a proportion of approximately 40% of all children from 2 to 6 years old from Santo Andre. We observed average monthly income per capita (AMPC) of 0.55 minimum wages (MW - ~U$175.00), the average number of persons per house was 4.6, the median years of mother schooling was 6 years and the median years of father schooling was 5 years. Half of the parents worked in trades such as unskilled and 15% were unemployed (Table 2).

**Table 1:** Children distribution according to gender and age

*Gender Age*	*Male n (%)*	*Female n (%)*	*Total n(%)*
***< 3 years***	69 (8.21)	71 (10.08)	140 (9.06)
***3 - 4 years***	251 (29.88)	179 (25.43)	430 (27.85)
***4.1- 4.9 years***	101 (12.03)	142 (20.18)	243 (15.75)
***> 5 years***	419 (49.88)	312 (44.31)	731 (47.34)

***Total***	840 (100)	704 (100)	1544 (100)

**Table 2:** Distribution of children parents enrolled in day care centers according to occupation*

*Ocupation*	*Father (%)*	*Mother (%)*
***Unemployed***	198 (19.1)	167 (11.5)
***Service generalities***	447 (43.1)	900 (62.2)
***Trade***	9 (10.5)	164 (11.3)
***Industries***	130 (12.5)	60 (4.1)
***Retired or pensioner***	20 (1.9)	7 (0.5)
***Others***	134 (13.0)	150 (10.4)
***Total***	1038 (100.0)	1448 (100.0)

*Adapted from the classification of Ministério da Fazenda - Secretaria da Receita Federal Natureza da Ocupação, www.receita.fazenda.gov.br

Weight (W) and height (H) were measured by researchers using internationally accepted techniques [19] under supervision of pediatricians from the daycare centers and all data were collected from records of child care using a standardized form. Anthropometric data were collected in the daycare center, according to Lohman et al [20] method. The child's weight was measured by using the Seca^® ^electronic scale with a split of 0.1 g, and height was measured by stadiometer wall (Seca^®^) with two meters and centimeters and millimeters subdivision [21]. In addition to weight and height we included the following variables: age, gender, birth weight, duration of breastfeeding, maternal age at child birth, level of maternal education, AMPC and how long they had attended the daycare center. Scales and stadiometers were calibrated regularly and reliably.

The values of W, H and body mass index (BMI) were transformed into z scores and percentiles based on the National Center for Health Statistics - 2000 (CDC/NCHS) framework and it was statistically analyzed in relation to the distribution and the association with socio-demographic and health data.

The cutoff points for nutritional disorders analysis were: -2 z scores and 2.5 and 10 percentiles for risk of malnutrition, 85 to 95 percentile for overweight and above the 95 percentile for obesity [22,23].

Data were stored in an Excel^® ^worksheet. In order to calculate anthropometric indices we used the software EpiInfo 3.3.2 Nutrition February 2005, which described the frequency distribution and central tendency measures (mean, standard deviation and median). Pearson's coefficient was used to evaluate the correlation between age and anthropometric indices.

The analysis was performed by the Stepwise Backward Regression method including the following variables: age, gender, birth weight, duration of breastfeeding, age of mother at birth and how long they had attended the daycare center which in univariate analysis showed a p < 0.15 in the evaluation of its association with excess weight (overweight and obesity, evaluated by z score of BMI). In order to verify if the proposed regression model fits the data well, we applied the F-test.

The level of significance (α) adopted for all statistical tests was 5%. The statistical processing of data was performed with SPSS ^® ^12.0.

## Results

Regarding the average growth achieved by the group of children as a whole, Table 3 shows that in the three variables studied: height, weight and BMI, the mean z score was above the reference values of the CDC/NCHS and that this difference was much higher with respect to weight and BMI. There was no significant difference between the variability of the variables collected in our study and the variability of the same variables according to the CDC/NCHS reference.

**Table 3:** Mean and standard deviation of z scores for height (height/age - HAZ), weight (weight/age - WAZ) and body mass index (BMI/age - BMIZ) according to gender in preschool children 2-6 years of age

*Gender*	*Male (n = 840)*	*Female (n = 704)*	*Total (n = 1544)*
***HAZ***	0.07 (1.039)	0.23 (0.99)	0.14 (1.02)
***WAZ***	0.28 (1.108)	0.33 (0.977)	0.30 (1.05)
***BMIZ***	0.41 (1.174)	0.41 (1.006)	0.41 (1.1)

In Table 4 the z scores for W and BMI tended to reach higher values than those of H in older children. Moreover, higher' values can also be observed in younger children: the z-score of BMI (or BMI/BMI/age) is higher than the z-score of H (or H/A - height/age) in children aged 2 years old as well. However, those differences did not reach statistical significance. The average values of z scores for W and H were higher in females, however, BMI were identical between the both genders. The slope of the three lines was statistically significant with F of 11.53 (p = 0.0426), 20.81 (p = 0.0197) and 27.70 (p = 0.0134), respectively, for W, H and BMI. The average H was the only variable in those younger than 3 years old below the median of the reference. The correlation coefficient for each of the 3 variables was quite high: 0.89, 0.93 and 0.95, respectively for W, H and BMI. Our findings indicate high prevalence of overweight and obesity affecting approximately one in four children.

**Table 4:** Mean and standard deviation of z scores of HAZ (height/age), WAZ (weight/age) and BMIZ (body mass index/age) of preschool children 2-6 years of age according to age

*Age*	*2 years (n = 141)*	*3 years (n = 313)*	*4 years (n = 354)*	*5 years (n = 453)*	*6 years (n = 278)*
***HAZ***	-0.154 (0.909)	0.072 (1.058)	0.081 (1.028)	0.165 (0.973)	0.183 (1.068)
***WAZ***	0.006 (1.052)	0.071 (1.111)	0.164 (1.004)	0.261 (1.031)	0.355 (1.042)
***BMIZ***	0.111 (1.189)	0.218 (1.245)	0.276 (1.06)	0.365 (1.014)	0.454 (1.032)

**Figure 1:**
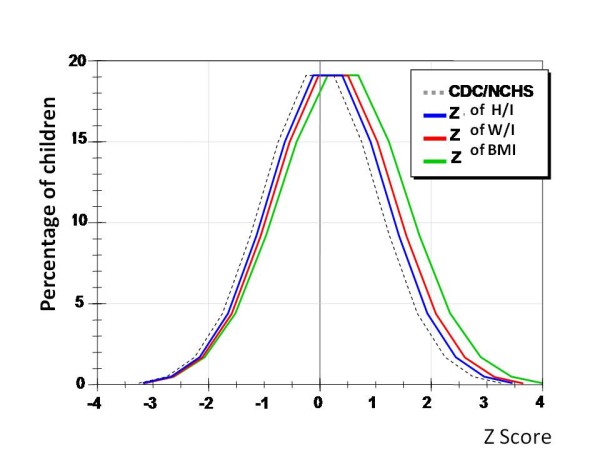
**Distribution of preschool children 2-6 years of age according to their z scores of weight, height and BMI for age**.

Child growth evaluation values in Figure 1 represented the distribution of z scores with respect to W, H, and BMI, which were shifted to the right of the standard, towards higher values, especially BMI. The average values of z scores of W and H were higher for girls, however, BMI were identical, suggesting no significant gender differences in the prevalence of obesity.

**Figure 2:**
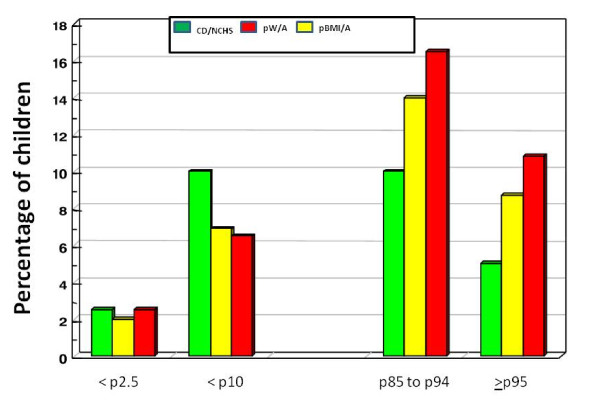
**Proportion of preschool children 2-6 years of age classified as nutritional risk according to percentiles of W/A (weight/age) and BMI/A (BMI/age)**. *The dark green bar represents the values according to the 2000 National Center for Health Statistics (CDC/NCHS).

In Figure 2, noting nutritional risks below 2.5 p or between 2.5 p and 10 p, for W and BMI, the proportion of children was decreased or equal compared to what was expected according to the CDC/NCHS reference, while the other spectrum extremity showed a higher than expected percentile proportion of obese or overweight values.

In Multinomial Logistic Regression, when using obesity as the only dependent variable, there was an association with girls and higher birth weight (3,000 g or more), (OR: 1.52; IC 95%: 1.17 to 2.12; p = 0.028 and OR: 1.8; IC 95%: 1.25 to -2.34; p = 0.0001, respectively). When analyzed together, obesity or overweight, as a single dependent variable, we observed only association with high birth weight, OR 1.44 (IC 95%: 1.19 to 2.01; p = 0.001).

## Discussion

Until recently the literature indicated that children from low income families presented growth development below their potential, with height values lower than the median values of the reference growth of children in industrialized countries, as proposed by the CDC/NCHS in the United States of America. These differences were observed among children from the same country or the same region differing only in socioeconomic class and it always reported the disadvantage of children from low income families [24]. The differences regarding height were the result of a worse nutritional status and not inadequate living conditions [25].

Daycare centers in Santo Andre showed growth in stature was similar and even higher, from four years old on, compared to the U.S. reference, even though they were children from low income families. We may propose some reasons for the prevalence of being overweight. First, this fact associated with low prevalence (or even absence) of malnutrition indicate improvement of living conditions of this population, particularly with regard to access to food and health care [4,26]. Second, our results indicated high prevalence of overweight and obesity, affecting approximately one in four children. The high prevalence to be overweight and obesity in children at preschool age was described in several countries, including Chile [27]. The anthropometric profile of preschool children found in Santo Andre is compatible with the rapid nutritional transition shown in developing countries, including Latin America [6]. Third, in these stages of epidemiological nutritional transition, daycare and preschools, which are traditionally regarded as a protective factor for malnutrition, might become a risk for overweight and obesity, since it is where children stay on average 8 hours per day, 5 days per week, and where about 75% of daily calories [28-30] provided. It is clear that this finding cannot be attributed directly and exclusively to daycare centers. Possibly low income families cannot access proper nutrition, which would account for the rest of their daily caloric intake. Moreover, it is possible that these pre-school children perform less physical activity at home, due to the use of television [3,29]. This situation may be considered as a risk factor for children born with normal or high weight [30]. Fourth, the interpretation of our findings indicates that overweight and obesity compromise growth at this stage of epidemiological and nutritional transition in low income children from an urban area of a developing country. This nutritional deficiency due to increased access to inadequate food quality tends to continue throughout life [31-33]. It results in elevated social costs to the health care system, which will financially affect these. All factors cited above may affect our population.

Our data are particularly alarming because obesity was already observed in 3 year old children with a tendency to increase as they grow. This trend may result in high rates of obesity in young adults, with a very early onset of morbidity [9,34]. The metabolic changes that occur as a result of rapid and intense growth tend to continue into itself until adulthood, hence compromising health [8].

Our study presents some points that should be addressed. Although a large part of the recognized classification systems of growth and development define preschool period from 3 to 6 years old [16,35] we investigated children from 2 to 6 years old. On the other hand, families in Brazil [36,37] do send 2 year old children to school. Not including maternal anthropometry is a limitation, because it does affect the children's nutritional status [38]. We did not use the outcome measure BMI-for-age percentile, as recommended by the CDC/NCHS for people aged 2-20. We used Z-scores only, the z-score indicates how many standard deviations an observation or data is above or below the mean [21]. We decided to use only z-score because it is our protocol.

## Conclusion

Main results showed that: 1) height, weight and BMI presented average z score are above the reference values of the CDC/NCHS, this difference was higher in weight and BMI; 2) z scores for weight and BMI tended to reach higher values than those of stature in older children; 3) the distribution of z scores with respect to weight, height and BMI were shifted towards higher values compared to the CDC/NCHS, more pronounced in BMI; 4) the average values of z scores of weight and height were higher in females, however, BMI were identical, suggesting no significant difference between gender regarding overweight prevalence and; 5) there was a percentile proportion of obese or overweight higher than expected in this population composed of children from low income families. In conclusion, preschool children from low income families are in an advanced stage of nutritional transition with a high prevalence for overweight and obesity.

## Competing interests

The authors declare that they have no competing interests.

## Authors' contributions

All authors participated in the acquisition of data and revision of the manuscript. DOS and CL conceived of the study, determined the design, performed the statistical analysis, interpreted the data and drafted the manuscript, VEV, LCA, VGN, PR, RW and AGO determined the design, interpreted the data and drafted the manuscript. All authors read and gave final approval for the version submitted for publication.
